# Targeting Patient-Derived Orthotopic Gastric Cancers with a Fluorescent Humanized Anti-CEA Antibody

**DOI:** 10.1245/s10434-024-15570-9

**Published:** 2024-06-18

**Authors:** Kristin E. Cox, Michael A. Turner, Thinzar M. Lwin, Siamak Amirfakhri, Kaitlyn J. Kelly, Mojgan Hosseini, Pradipta Ghosh, Marygorret Obonyo, Robert M. Hoffman, Paul J. Yazaki, Michael Bouvet

**Affiliations:** 1 Department of Surgery, University of California San Diego, La Jolla, CA USA; 2 VA San Diego Healthcare System, San Diego, CA USA; 3 Department of Surgical Oncology, City of Hope National Medical Center, Duarte, CA USA; 4 Department of Surgical Oncology, University of Wisconsin, Madison, WI USA; 5 Department of Pathology, University of California San Diego, La Jolla, CA USA; 6 Department of Cellular and Molecular Medicine, University of California San Diego, La Jolla, CA USA; 7 Department of Medicine, University of California San Diego, La Jolla, CA USA; 8 AntiCancer Inc, San Diego, CA USA; 9 Department of Immunology & Theranostics, Beckman Research Institute of the City of Hope, Duarte, CA USA

**Keywords:** Gastric cancer, Patient-derived orthotopic xenograft, PDOX, Fluorescence, Fluorescent antibody, CEA, Tumor targeting, Tumor labeling

## Abstract

**Background:**

Gastric cancer poses a major diagnostic and therapeutic challenge as surgical resection provides the only opportunity for a cure. Specific labeling of gastric cancer could distinguish resectable and nonresectable disease and facilitate an R0 resection, which could improve survival.

**Methods:**

Two patient-derived gastric cancer lines, *KG8* and *KG10*, were established from surgical specimens of two patients who underwent gastrectomy for gastric adenocarcinoma. Harvested tumor fragments were implanted into the greater curvature of the stomach to establish patient-derived orthotopic xenograft (PDOX) models. M5A (humanized anti-CEA antibody) or IgG control antibodies were conjugated with the near-infrared dye IRDye800CW. Mice received 50 µg of M5A-IR800 or 50 µg of IgG-IR800 intravenously and were imaged after 72 hr. Fluorescence imaging was performed by using the LI-COR Pearl Imaging System. A tumor-to-background ratio (TBR) was calculated by dividing the mean fluorescence intensity of the tumor versus adjacent stomach tissue.

**Results:**

M5A-IR800 administration resulted in bright labeling of both *KG8* and *K10* tumors. In the *KG8* PDOX models, the TBR for M5A-IR800 was 5.85 (SE ± 1.64) compared with IgG-IR800 at 0.70 (SE ± 0.17). The *K10* PDOX models had a TBR of 3.71 (SE ± 0.73) for M5A-IR800 compared with 0.66 (SE ± 0.12) for IgG-IR800.

**Conclusions:**

Humanized anti-CEA (M5A) antibodies conjugated to fluorescent dyes provide bright and specific labeling of gastric cancer PDOX models. This tumor-specific fluorescent antibody is a promising potential clinical tool to detect the extent of disease for the determination of resectability as well as to visualize tumor margins during gastric cancer resection.

Gastric cancer is the fifth most common cancer diagnosed and is the fourth leading cause of cancer-related deaths worldwide, with the highest rates found in Eastern Asia and Eastern Europe.^1^ In the United States, gastric cancer is the fifteenth most commonly diagnosed cancer, and recent evidence suggests that the incidence of early-stage gastric cancer is increasing.^2,3^ Current guidelines by the National Cancer Comprehensive Network (NCCN) and European Society for Medical Oncology (ESMO) recommend resection for localized gastric cancers, with the possibility to perform endoscopic resection for select small T1a tumors.^4,5^ In these gastric cancers surgeries, the ability to achieve an R0 resection (negative margins) is the single most important factor for improving outcomes. A metanalysis of more than 10,000 patients from 14 studies demonstrated that patients who had a R1 resection had an overall survival hazard ratio of 2.06 compared with patients who received an R0 resection.^6^

Fluorescence-guided surgery (FGS) has emerged as a useful adjunct in oncologic resections to visualize tumor deposits and aid in their removal. The efficacy of antibodies conjugated to NIR fluorophores to label and enhance the detection of breast, lung, pancreatic, prostate, and colorectal cancers have been demonstrated in preclinical mouse models.^7–20^ Several of these studies have now been translated to human trials, which have shown that the use of tumor-specific fluorescence labeling can detect additional residual tumor deposits or previously unrecognized synchronous disease in 14–50% of cases.^21–25^ However, there are currently no FDA-approved agents for FGS of gastric cancer. Given the impact that an R0 resection has on survival outcomes, there is a critical need for development of agents to label gastric cancer.

One potential tumor-specific target is carcinoembryonic antigen (CEA), as 74.5–90% of gastric cancers have been shown to express CEA by immunohistochemistry.^26,27^ We have previously utilized the poorly differentiated gastric adenocarcinoma cell line, MKN45, to create orthotopic mouse models of gastric cancer. Using a humanized anti-CEA antibody (M5A) conjugated with a NIR 800 nm dye (M5A-IR800), we were able to brightly target the tumors and achieve high tumor-to-background ratios.^28^ In the present study, we obtained two patient-derived gastric cancer samples and demonstrate the applicability of M5A-IR800 to target human gastric cancer.

## Methods

### Mouse Models

All studies were approved by the San Diego Veterans Administration Medical Center Institutional Animal Care and Use Committee (IACUC) animal-use protocol A17-020 and the University of California San Diego (UCSD) IACUC protocol S99001. Athymic male and female nude mice, aged 4–6 weeks were purchased from the Jackson Laboratory (Bar Harbor, ME). The animals were fed an autoclaved diet and housed in a barrier facility. Orthotopic mouse models were fed a chlorophyll-reduced diet for 2 weeks before imaging (Envigo, Indianapolis, IN) to reduce autofluorescence. Before any surgical procedure, the mice were anesthetized with a solution of xylazine, ketamine, and phosphate-buffered saline (PBS) via intraperitoneal injection. This solution was used, because it provides stable anesthesia without the need to titrate for depth of sedation for the duration of the surgical procedure. For postoperative pain control, they received subcutaneous buprenorphine reconstituted in PBS (dosage: 0.05 mg/kg). At the conclusion of the study, mice were anesthetized with isoflurane and euthanized by cervical dislocation.

### Patient-Derived Gastric *Cancer* Xenografts

The patient-derived gastric cancers *KG8* and *KG10* were obtained from surgical specimens under sterile conditions at the time of surgical resection. The patients’ tumors were obtained with informed consent under the UCSD Institutional Review Board (IRB) protocol number 090401.

### Xenograft Establishment

To initially establish *KG8* and *KG10* mouse models, 1 mm^3^ fragments of the patient’s tumor were implanted into the bilateral flanks and shoulders of nude mice. Once subcutaneous tumors grew to approximately 1 cm, subsequent passages were performed by harvesting 1 mm^3^ fragments and implanting them into new mice. In additional nude mice, patient-derived orthotopic xenograft (PDOX) models were established by using the method of surgical orthotopic implantation described by Furukawa et al.^29^ In brief, mice were anesthetized as described above and a 1–2 cm transverse incision was made in the left upper quadrant through which the stomach was delivered. Subcutaneous-grown tumors were harvested and ~1-mm^3^ fragments were affixed to the greater curve of the stomach using 8–0 nylon suture (Ethicon Inc., Somerville, NJ). The stomach was returned to the abdomen and the incision was closed with interrupted 6-0 vicryl sutures (Ethicon Inc.). Orthotopic models were allowed to grow for 4–6 weeks for *KG8* and 6–10 weeks for *KG10* before performing any imaging studies. An equal distribution of male and female mice was used for all experiments.

### Antibody Conjugation

The humanized anti-CEA hT84.66-M5A (M5A) monoclonal antibody, established by Yazaki et al., was used for labeling of the gastric cancers.^30^ A control antibody that binds the heavy chains on human IgG was used as a control (R&D Systems, Minneapolis, MN). Both M5A and IgG were conjugated to the NIR dye IRDye800CW NHS Ester (LI-COR Biosciences, Lincoln, NE) to establish M5A-IR800 and IgG-IR800 by using methods previously described.^18,31^ The final products were stored at 4 °C.

### Antibody-conjugate Administration and Imaging

50 µg micrograms of M5A-IR800 and 50 µg of IgG-IR800 were made by diluting the compounds in PBS for a total injection volume of 100 µl. The antibodies were administered via tail vein injection to mice bearing PDOX tumors. After 72 hr, the mice were euthanized, and a laparotomy was performed to allow imaging of intra-abdominal tissues. Imaging was performed with the Pearl Trilogy Small Animal Imaging System (LI-COR Biosciences, Lincoln NE) with 800-nm wavelength excitation.

### Imaging and Data Processing

All images within the *KG8* or *KG10* orthotopic models treated with either M5A-IR800 or IgG-IR800 were linked, and the same brightness and contrast settings were used for all images in the present study. Within the Pearl Trilogy Small Animal Imaging System software, analysis circles were drawn around the tumors and background tissue (adjacent normal stomach) while viewing the bright light images. Fluorescence signal quantification was performed by using the LICOR Image Studio software. The mean fluorescence intensity of the 800-nm signal was measured for each region of interest and tumor-to-background ratios (TBR) were calculated.

### Statistical Analysis

Statistical analysis was performed by using R software (Free Software Foundation, Boston, MA). Data from both the *KG8* and *KG10* experiments were found to be normally distributed by using the Shapiro test. A Student’s *t*-test with two tails was performed to compare the TBRs of M5A-IR800 versus IgG-IR800 in the orthotopic gastric cancer models. A *p*-value of < 0.05 was used as a predetermined cutoff for statistical significance.

### Immunohistochemistry

Tumor samples were removed en bloc with surrounding tissue at the time of mouse necropsy. Samples were fixed in formalin for at least 72 hr before being embedded in paraffin and sectioned. Slides were stained with hematoxylin and eosin (H&E) per standard protocols. An experienced pathologist (MH) performed interpretation of the histologic slides.

## Results

### Patient-Derived Gastric *Cancer* Specimens 

**Fig. 1:**
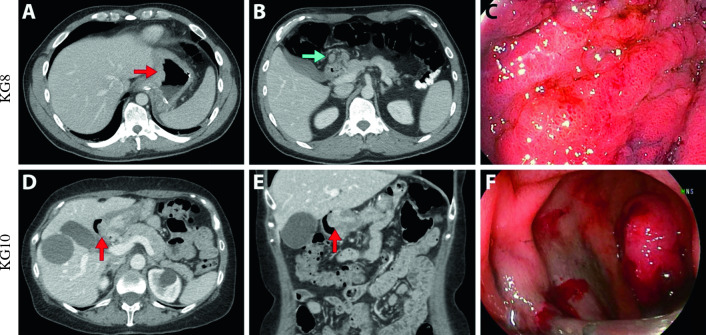
Patient-derived gastric cancers *KG8* and *KG10*. **A** Axial computerized tomography (CT) images of gastric cardia thickening. **B** Axial CT images with enlarged perihepatic lymph nodes. **C** Endoscopic images showing circumferential friable tumor extending from cardia to antrum consistent with linitis plastica. **D** Axial and **E** coronal CT images demonstrating ~3 cm polypoid mass at pylorus. **F** Endoscopic images demonstrating an obstructing mass. Red arrow: tumor, blue arrow: enlarged lymph node

Tumor specimens were obtained from two patients who underwent gastrectomy for gastric cancer (Table 1). The first, *KG8*, was a 45-year-old male who presented with early satiety and weight loss. Imaging revealed diffuse thickening of the stomach and numerous enlarged lymph nodes (Fig. 1A–B) and an elevated serum CEA of 176. Esophagogastroduodenoscopy (EGD) revealed circumferential friable tumor consistent with linitis plastica (Fig. 1C). Given the patient’s inability to tolerate enteral feeding, upfront surgery was performed. *KG10* was obtained from a 66-year-old female with a history of a Roux-en-Y gastric bypass who presented with epigastric pain. Imaging and EGD demonstrated an obstructing polypoid-type mass in the gastric remnant (Fig. 1D–F), although serum CEA was normal at 5.7. Although the patient was scheduled to receive neoadjuvant therapy prior to resection, gastrointestinal bleeding from the tumor necessitated upfront surgical resection.

**Table 1:** Patient demographics for the patient-derived gastric cancer lines *KG8* and* KG10*

	Age	Gender	Tumor location	Differentiation	Stage	CEA level (ng/mL)
*KG8*	45	Male	Diffuse thickening	Well-to-moderately differentiated	pT4aN3bM1	176
*KG10*	66	Female	Polypoid mass at pylorus	Poorly differentiated	pT4aN3b	5.7

### Bright and Specific Labeling of Orthotopic KG8 Tumors

**Fig. 2:**
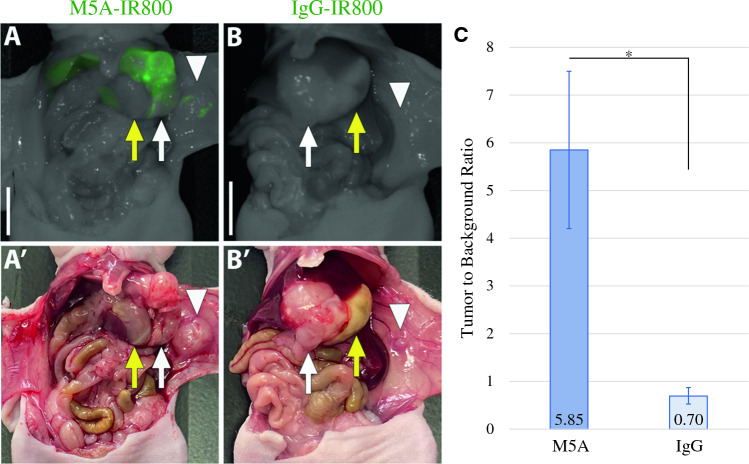
Fluorescence labeling of *KG8* orthotopic gastric tumors. **A** M5A-IR800 brightly labels primary gastric tumor. **A’** Bright light imaging with a gastric tumor seen on the greater curvature of the stomach. **B** Lack of gastric tumor labeling with IgG-IR800. **B’** Bright light imaging with a gastric tumor seen directly invading liver parenchyma. White arrow: tumor, yellow arrow: stomach, arrowhead: abdominal wall metastasis. Scale bar: 1 cm. **C** Average TBRs of gastric tumors labelled with M5A-IR800 or IgG-IR800. Error bars represent standard error. *p-value: 0.035

Tumor fragments from the *KG8* surgical specimen were implanted into the flanks of nude athymic mice. Once the tumors reached adequate size, they were harvested, and 1-mm^3^ fragments were affixed to the greater curvature of the stomach in additional nude mice to establish patient-derived orthotopic xenograft (PDOX) models. After 4–6 weeks, mice were randomized to receive either M5A-IR800 or the control (IgG-IR800) intravenously. Fluorescence labeling with M5A-IR800 resulted in bright targeting of the *KG8* orthotopic gastric cancers compared with control after 72 hr (Fig. 2A–B’). A mean TBR of 5.85 (±1.64) was seen for M5A-IR800 (*n* = 5) compared with 0.70 (±0.17) for the control (*n* = 4) with a *p*-value of 0.035 (Fig. 2C).

### Bright and Specific Labeling of Orthotopic KG10 Tumors

**Fig. 3:**
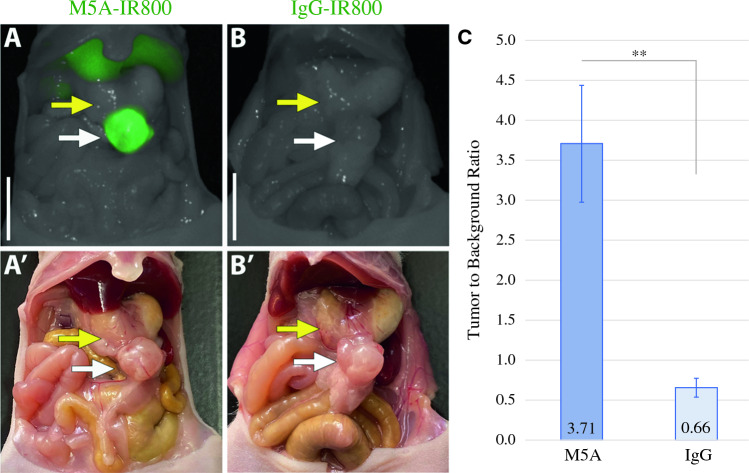
Fluorescence labeling of *KG10* orthotopic gastric tumors.** A** M5A-IR800 brightly labels a primary gastric tumor. **A’** Bright light imaging with a gastric tumor seen on the greater curvature of the stomach. **B** Lack of gastric tumor labeling with IgG-IR800. **B’** Bright light imaging with a gastric tumor seen on the greater curvature of the stomach. White arrow: tumor, yellow arrow: stomach. Scale bar: 1 cm. **C** Average TBRs of gastric tumors labelled with M5A-IR800 or IgG-IR800. Error bars represent standard error. *p*-value: 0.009

The same process of establishing PDOX models was used for the *KG10* line. Bright labeling of the *KG10* PDOX models also was seen with M5A-IR800 compared with control (Fig. 3A–B’). Labeling with M5A-IR800 (*n* = 6) resulted in an average TBR of 3.71 (±0.73), whereas those labeled with IgG-IR800 (*n* = 5) had a mean TBR of 0.66 (±0.12) with a *p*-value of 0.009 (Fig. 3C).

### Immunohistochemistry of Patient-Derived Gastric *Cancer* Lines

**Fig. 4:**
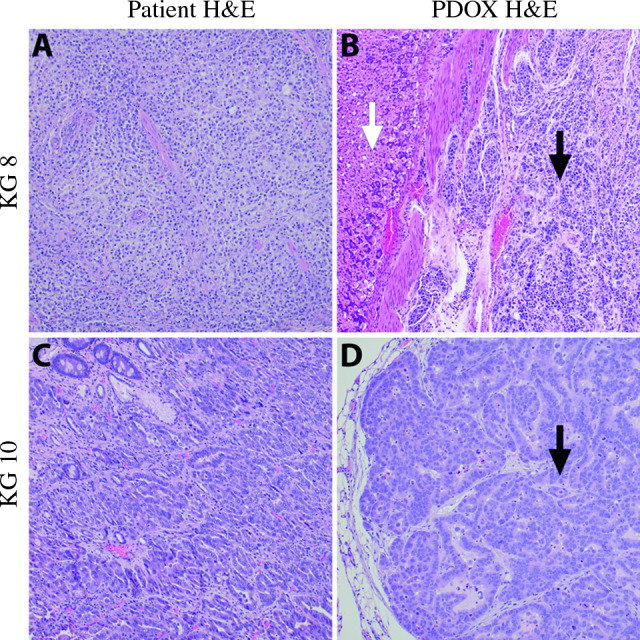
Pathology of *KG8* and *KG10* patient-derived gastric cancer lines. **A** H&E of the *KG8* patient’s original pathology from surgical resection showing poorly differentiated gastric adenocarcinoma. **B** H&E of the *KG8* patient-derived orthotopic xenograft tumor with poorly differentiated cells denoted by black arrow and normal tissue with white arrow. **C** H&E of the *KG10* patient’s original pathology from surgical resection showing well-to-moderately differentiated gastric adenocarcinoma. **D** H&E of the *KG10* patient-derived orthotopic xenograft tumor with well-to-moderately differentiated cells denoted by black arrow.

For *KG8*, hematoxylin and eosin (H&E) staining of the patient’s surgical specimen and the PDOX tumor demonstrated poorly differentiated adenocarcinoma (Fig. 4A and B). H&E staining of the *KG10* patient’s surgical specimen and the *KG10* PDOX tumor demonstrated well-to-moderately differentiated adenocarcinoma (Fig. 4C and D). These results demonstrate that the PDOX models retained the pathologic characteristics of the original donor patients’ tumors.

## Discussion

In gastric cancer, the only opportunity for cure is with a R0 resection as perioperative chemotherapy has provided only modest improvements in overall survival.^32,33^ The field of fluorescence-guided surgery (FGS) has emerged as an opportunity to address this need for improved surgical resections. There has been a growing emergence of the use of targeted fluorescence with tumor-specific markers for many cancer types.^34^ While earlier work in the field often used fluorescent dyes in the visible spectrum, such as 488 or Cy5, most work now focuses on the use of NIR fluorophores as they have increased tissue depth penetration, reduced light scattering, and reduced autofluorescence.^34,35^ In recent years, 5-ALA and Cytalux have gained FDA approval for the fluorescence-guided surgical resection of gliomas and ovarian and lung cancer, respectively.^21,36^ There are ongoing clinical trials for the use of an anti-CEA antibody conjugated to a 700 nm dye (SGM-101) in both colorectal cancer and pancreatic cancer.^37^

In gastric cancer, a few probes have been evaluated for fluorescence labeling of tumors in preclinical models, although currently, none are FDA-approved for use in FGS.^38^ Hoetker et al. tested Cetuximab and another anti-EGFR antibody, bound to FITC and Alexa Fluor 488 respectively, with confocal laser endomicroscopy (CLE) and showed increased fluorescence in MKN45 tumors compared with isotype-control antibody.^39^ MG7, a novel tumor-associated antibody originally made by Fan et al. by inoculating mice with the MKN46-9 gastric cancer cell line, also has been tested for fluorescence labeling of gastric cancer.^40^ MG7 was labeled with Alexa Fluor 680, and using CLE, showed increased fluorescence in xenograft tumors (BGC-823 and SGC-7901 cell lines) compared with a control antibody.^41^ There are significant challenges with probe selection for gastric cancer; EGFR expression is only seen in 62% of gastric cancers, and although MG7 is expressed in 94% of gastric cancers, it also is seen in *Helicobacter pylori*-associated gastritis.^42–44^

Koga et al. utilized an anti-CEA antibody labeled with Alexa Fluor 594 to label orthotopic mouse models of MKN45 gastric cancer. Although their probe was able to visualize the tumors, background signals (signals from surrounding normal tissue) were high.^45^ Despite the limited utility of serum CEA levels to detect gastric cancer upon initial diagnosis or at the time of recurrence, 74.5–90% of gastric cancers have been shown to express CEA by immunohistochemistry.^26,27,46–48^ Therefore, CEA is an excellent target for fluorescence labeling of gastric cancer.

Previously, we utilized the MKN45 cell line to establish orthotopic mouse models of gastric cancer and labeled them with a humanized anti-CEA antibody (M5A) conjugated with a NIR 800 nm dye (M5A-IR800).^28^ Our findings using the cell line demonstrated that the conjugate was able to brightly label both primary gastric tumors and peritoneal metastases with TBRs greater than four times that of the control. In the present study, we evaluated this probe in patient-derived orthotopic xenograft (PDOX) models. Compared with the homogeneous nature of human cancer cell lines, patient-derived xenografts more closely mimic the heterogeneity of patient tumors.^49^ Additionally, the metastatic pattern of PDOX models has been shown to correlate to the pattern of disease spread in the donor patients.^50^ Thus, we evaluated whether our probe could maintain a high level of fluorescence intensity and contrast given tumor heterogeneity. Two gastric cancer specimens (*KG8* and *KG10*) were obtained from patients undergoing surgical resection to establish PDOX models of gastric cancer. We showed that M5A-IR800 selectively and brightly labeled multiple patient-derived gastric tumors with high TBRs irrespective of the patient’s preoperative serum CEA levels.

Limitations of the study include the use of nude athymic mice and the location of the tumors. To address the use of immunocompromised mice, future studies could include testing our probe in a transgenic mouse that expresses human CEA as a syngeneic model of gastric cancer.^51^ Regarding tumor location, our current method of attaching tumor fragments to the serosal surface of the mouse stomach has room for improvement as most gastric cancers are intraluminal and often infiltrative within the layers of the gastric wall. To address this aspect of gastric cancer pathophysiology, we are developing improved models to achieve better incorporation of the tumors into the layers of the stomach.

Additionally, fluorescence labeling of lymph nodes containing metastatic disease is a provocative area of study that we are currently working on with our new models. There have been numerous studies investigating the role of indocyanine green (ICG) in detecting sentinel lymph nodes, nodes containing metastatic disease, or the improved detection and thus completion of a D2 lymphadenectomy.^52–59^ Despite the robust body of research on the use of ICG in gastric cancer lymphadenectomy, its use has yet to become incorporated into the current guidelines or to become the standard of care.^60^ This is likely due to the complex lymphatic drainage of the stomach and nonspecific nature of ICG. A tumor-specific probe given systemically could overcome these challenges encountered with ICG’s ability to detect lymph nodes containing metastatic disease. If M5A-IR800 can label lymph nodes containing metastatic disease in addition to the primary tumors, it would drastically increase the value of using the probe during surgical resection.

In addition to testing M5A-IR800 on the new model of gastric cancer and metastatic lymph nodes, other areas of further research include performing FGS on orthotopic mouse models of gastric cancer and monitoring for recurrence or improvement in overall survival. In previous work on pancreatic cancer using an anti-CEA antibody conjugated to Alexa Fluor 488, median disease-free survival for the FGS group was 11 weeks compared with 5 weeks for the bright-light surgery group.^61^ The enhanced ability to visualize the gastric tumors at the time of surgery should result in improved resections and thus survival, although studies are needed to test this hypothesis. Other future directions could include optimizing the dye to which the antibody is conjugated. There are numerous imaging devices currently used in the operating room, many of which have slight variations in their optimal NIR window, which could necessitate tuning of NIR dyes to specific clinical devices.^62^

Additional applications of tumor-specific probes for gastric cancer include its use in positron emission tomography (PET) imaging for either the initial diagnosis or monitoring for disease recurrence. Xu et al. utilized MG7 and labeled it with Gallium-68 for PET imaging of mice bearing subcutaneous tumors.^63^ Unfortunately, MG7 expression also is seen in *H. pylori* associated gastric disease, which could limit its use in clinical practice.^44^ Cadherin-17 labeled with Indium-111 also was used for PET imaging of subcutaneous tumor-bearing mice and showed excellent specificity for the tumors, although only 64% of gastric cancers express Cadherin-17.^64^ Trastuzumab, the monoclonal antibody against HER2, has been used for PET imaging of gastric cancer by labeling with Zirconium-89 or Copper-64.^65,66^ Although Trastuzumab is already FDA-approved and provides the potential for both treatment and enhanced imaging when coupled with PET radiotracers, only 20% of gastric cancers express HER2.^67^

M5A also has been used for PET imaging of many CEA-positive cancers, including colorectal cancer (*n* = 11), medullary thyroid cancer (*n* = 5), esophagogastric cancer (*n* = 2), and pancreatic cancer (*n* = 2).^68^ In a phase 1 trial of M5A labelled with Yttrium-90, it was shown that the humanized version of M5A led to decreased risk of immunogenicity (development of human antibodies against a drug or other molecule) compared with previous work with chimeric versions of M5A.^69^

There are many potential clinical applications for tumor-specific markers as described above. In the present study, we investigated the use of an anti-CEA antibody conjugated to a NIR dye for fluorescence labeling of gastric cancers in clinically relevant PDOX models. These results demonstrate its potential for future clinical applications in targeted fluorescence-guided surgery.
